# Clinical Characteristics of Patients Undergoing D2 Gastrectomy with Bursectomy in Gastric Cancer Patients: An Observational Study

**DOI:** 10.31729/jnma.v63i290.9209

**Published:** 2025-09-01

**Authors:** Sundar Shrestha, Jainendra Kumar Chaudhary, Sharad Pokhrel, Indra Kumar Jha, Niroj Banepali, Samyukta K.C.

**Affiliations:** 1 Department of Surgical Gastroenterology, Bir Hospital, National Academy of Medical Sciences, Kathmandu, Nepal

**Keywords:** *bursectomy*, *gastric cancer*, *tumor deposits*

## Abstract

**Introduction:**

Gastric cancer is a significant global and national health problem with high incidence and mortality rates. Despite advancements in treatment, local recurrence, mainly peritoneal, is common. Bursectomy, once the standard of surgery in resectable GC to reduce recurrence by removing the peritoneal membranes’ micro-metastasis, poses risks like pancreatic injury. Japanese guidelines now advise against it due to limited survival benefits, recommending D2 dissection with omentectomy instead. However, bursectomy is still routinely practiced in Nepal. This study evaluates its relevance, risks, and compliance with global guidelines.

**Methods:**

This descriptive cross-sectional study was conducted at Bir Hospital from March 2024 to May 2025, involving 35 patients with resectable stage Ib—III gastric cancer who underwent D2 gastrectomy with bursectomy. Ethical approval was obtained from NAMS IRB. Patients with poor performance status or metastasis were excluded. All procedures were performed by experienced consultant surgical gastroenterologists, and specimens were sent separately for examination.

**Results:**

Among 39 selected patients, 35 underwent curative D2 gastrectomy with bursectomy. Among them, 20 (57.14%) were male, with a mean age of 60.54±13.81 years and performance status 0-2. 33 (94.28%) patients had distal gastric lesions; among them, 27 (77.13%) patients had cT3+cT4 lesions. Tumor deposits in the bursa were seen in 3(8.57%) patients. There were procedure-specific complications like 2(5.71%) biochemical leaks, delayed 4(11.43%) gastric emptying, and 3(8.57%) postoperative ileus. There was no mortality. 18 (51.42%) patients were followed up for more than 6 months.

**Conclusions:**

There are tumor deposits in the bursa, and bursectomy doesn’t increase the post-operative complications.

## INTRODUCTION

Gastric cancer (GC) is significant global health issue, with over one million new cases annually. It is the third leading cause of cancer-related deaths worldwide and the fourth most common cause of mortality among cancers in Nepal.^1^ Despite radical surgery and perioperative chemotherapy, advanced gastric cancers have a recurrence rate of 30-40%.^2^ Surgery remains the most effective treatment, although it is not without limitations.

Bursectomy, removal of the peritoneal membrane over the transverse mesocolon and pancreas, aims to eliminate microscopic tumor deposits and reduce the risk of peritoneal metastasis.^3^ It was previously recommended by the Japanese Gastric Cancer Association for advanced gastric cancers.^4^ However, the procedure carries risks, including postoperative pancreatic fistula, delayed gastric emptying, and intestinal obstruction, which have raised concerns about its safety and benefit.^5,6^

The recent Japanese guidelines no longer recommend bursectomy due to insufficient evidence for survival benefit.^7^ Despite this shift, bursectomy remains routine in many Asian countries. This study examines prevalence of tumor deposits in bursa and associated complications.

## METHODS

This is an observational cross-sectional study performed in the department of Surgical Gastroenterology, Bir Hospital, National Academy of Medical Sciences (NAMS). The population for the study consisted of patients with resectable gastric carcinoma who underwent D2 gastrectomy with bursectomy from March 2024 to May 2025. Ethical approval was taken from the Institutional Review Board (IRB) of NAMS (Reference number: 1170/2080081)

All patients with resectable stage Ib to stage III (cT1 to cT4a) gastric cancer were included. Patients with poor performance status (Eastern Cooperative Oncology Group, ECOG ≥ 3, American Society of Anesthesiologists, ASA > 3), metastatic gastric cancer, visible tumor deposits on omentum and adjacent organs were excluded.

Bursectomy was performed by removing the peritoneal lining of bursa omentalis from the anterior layer of the transverse mesocolon up to the left gastroepiploic artery (4sb) and the anterior surface of the pancreas to a greater extent, up to the distal half of the pancreatic tail in case of distal gastrectomy. In case of total or proximal gastrectomy, the bursectomy extends up to the edge of the left lobe of the liver, excluding the distal half of the pancreatic tail. The specimen was sent in a separate specimen container. Surgery was performed by a consultant surgical gastroenterologist.

Clinical staging of the tumor was done according to the American Joint Committee for Cancer (AJCC) staging.^8^ All the resectable gastric cancer patients underwent D2 gastrectomy with bursectomy as per the institutional protocol following standard technique. Post-operative Pancreatic Fistula (POPF) was labelled if the drain fluid amylase level was more than three times the upper limit of the institutional normal value (i.e.,>300 IU/L) on or after Post Operative Day three (POD3).^9^ Delayed Gastric Emptying (DGE) was defined as failure to return to standard diet by the end of the first week POD.^10^ Blood loss was measured using the standard protocol.^11^ Postoperative morbidity or complications were defined as any deviation from the normal postoperative course and graded according to the Clavien-Dindo classification.^12^ Surgical site infection was defined according to the Centers for Disease Control and Prevention (CDC) guidelines in 2023 and classified as superficial, deep, or organ/ space infection.^13^ Patients were followed up for a minimum of one month to 11 months; 18 patients were followed up for at least six months. Patients received Neoadjuvant Chemotherapy (NACT), either FLOT (5-fluorouracil, leucovorin, oxaliplatin and docetaxel) or FOLFOX (folinic acid, 5-fluorouracil and oxaliplatin) as decided by the oncologist.

Statistical analysis was done using Statistical Package for Social Sciences (SPSS) v. 26 sofwtare by descriptive statistics.

## RESULTS

Out of the total 39 patients selected for the study, 35 underwent curative D2 gastrectomy with omentectomy and bursectomy, and four patients were excluded from the study for various reasons (two for laparoscopic-assisted gastrectomy with no bursectomy, one for gastrectomy combined with pancreaticoduodenectomy, and the other for inadequate bursectomy).

Out of the total 39 patients selected for the study, 35 (89.75%) patients underwent curative D2 gastrectomy with omentectomy and bursectomy, and 4 (10.25%) patients were excluded from the study; 2 for laparoscopic-assisted gastrectomy with no bursectomy, 1 for gastrectomy combined with pancreaticoduodenectomy, and the other one for inadequate bursectomy. The mean age ofthe patients was 60.54±13.81 years. Among them, 33 (94.29%) patients presented with pain, and 11 (31.43%) presented with associated complications, 6 (17.14%) with GOO, and 4 (11.43%) with bleeding requiring multiple units of blood transfusions. A total of 28 (80.00%) patients were smokers, 12 (34.29%) had blood group A positive, 6 (17.14%) patients received NACT, 4 (11.43%) received FLOT, and 2 (5.71%) received FOLFOX. A total of 25 (71.43%) patients had a performance status, ECOG 1 (Eastern Cooperative Oncology Group) and 20 (57.14%) patients had an ASA 2 (American Society of Anesthesiologists) due to associated comorbidities, complications, and nutritional status of the patients (Table 1).

**Table 1:** Characteristic of patients with resectable gastric carcinoma who underwent D2 gastrectomy with bursectomy (n=35).

Variables	Total n(%)
**Gender**	
Male	20(57.14)
Female	15(42.86)
**Symptoms**	
Pain	33(94.29)
Pain + other	19(54.29)
Gastric Outlet Obstruction (GOO)	6(17.14)
Bleeding	4(11.48)
**Habits**	
Smoking	28(80.00)
Alcohol	13(37.14)
Salted foods	25(71.43)
**NACT Regimen**	
FLOT	4(11.48)
FOLFOX	2(5.71)
**ECOG Status**	
ECOG 0	8(22.86)
ECOG 1	25(71.43)
ECOG 2	2(5.71)
**ASA Score**	
ASA I	15(57.14)
ASA II	20(42.86)

GOO = Gastric Outlet obstruction, ECOG = Eastern Cooperative Oncology Group, ASA = American Society of Anesthesiologists, NACT = Neoadjuvant Chemotherapy, FLOT = 5-fluorouracil, leucovorin, oxaliplatin, and docetaxel, FOLFOX = folinic acid, 5-fluorouracil and oxaliplatin.

A total of 33 (94.29%) patients underwent D2 distal gastrectomy, and 2 (5.71%) underwent D2 total gastrectomy with curative intent. Bursectomy was done in all of these patients following the standard surgical technique, both in distal and total gastrectomy.

In 33 (94.28%) patients, the tumor was located distally; out of them, 18 (51.4%) were distal and circumferential. Mean operative time was 229.09 ± 45.525 minutes (range: 180 - 460 minutes). Median lymph node yields were 16.00 (IQR:12 - 19), out of which the median positive lymph node rate was 2 (IQR:0- 4). Mean blood loss was 246.57 ± 82.816 ml (range: 150 - 450 ml). The mean post-operative hospital stay was 10.74 ±3.551 days (Table 2).

**Table 2:** Surgical profile of patientswith resectablegastric carcinoma who underwent D2 gastrectomy withbursectomy (n=35).

Variables	Total n (%)
**Types of Gastrectomy**	
D2 Distal	33(94.29)
D2 Total	2(5.71)
**Location of Lesion**	
Distal and circumferential	17(48.57)
Distal and posterior	13(37.17)
Distal and anterior	3(8.57)
Proximal	2(5.71)

There was a total of 19 (54.28%) cT3 tumors, and 8 (22.85%) cT4 tumors. Tumor deposits in the bursa (bursa positive) were seen in 3 (8.57%) cases. Among them, 2 had T4 lesions and 1 had a T3 lesion, altogether accounting for 3 out of 27 (11.11%) patients in the cT3/cT4 group in the subgroup analysis. All of them were distal stomach lesions with circumferential involvement of the stomach lumen. Histologic grades of 16 (45.71%) patients were poorly differentiated. 3 (18.75%) bursa-positive patients had poorly differentiated histology types in subgroup analysis. In 2 (5.7%) patients who received neoadjuvant chemotherapy (NACT), 1 received FLOT and the other received FOLFOX. Complete response (no residual tumor cells) with fibrosis was found in the final histopathological examination of the specimens of these 2 patients (Table 3).

**Table 3:** Bursa status and histological grade of patients with resectable gastric carcinoma who underwent D2 gastrectomy with bursectomy (n=35).

Variables	Total N (%)	Bursa Positive n(%)	Bursa Negative n(%)
**cT Stage**			
cT2	6(17.14)	0	6 (17.1)
cT3	19(54.28)	1 (5.26)	18 (94.74)
cT4	8(22.85)	2 (25.00)	6(75.00)
Final Histological Grade			
Poorly Differentiated	16(45.71)	3 (18.75)	13(81.25)
Moderately Differentiated	14(40.00)	0	14(100)
Well Differentiated	3(8.57)	0	3(100)
Complete Response	2(5.71)	0	2(100)

Surgical site infection (SSI) was seen in 12(34.29%) patients as a common post-operative complication. All of them had Clavien-Dindo Grade <IIIa complications. 4 (11.43%) patients had delayed gastric emptying (DGE), 3 (8.57%) had post-operative ileus and 2 (5.71%) patients had post-operative NG bleeding.

None of them required a blood transfusion. 2 (5.71%) patients had biochemical leak with raised drain fluid amylase levels of 342 and 319 mg/dl; none of them had clinically relevant postoperative pancreatic fistula (CR-POPF). 3 (8.57%) patients were readmitted; 1 (2.85%) for recurrent vomiting and 2 (5.71%) for chest infections. 18 (51.42%) patients were followed up for more than six months (Table 4).

**Table 4:** Post-operative morbidity of patients with resectable gastric carcinoma who underwent D2 gastrectomy with bursectomy (n=35).

Variables	Total n(%)	Clavien Dindo
**SSI**		
Superficial	12(34.29)	I
**Chest Complications**		
Atelectasis	3(8.57)	I
Effusions	6(17.14)	II
Consolidations	2(5.71)	II
Post-operative ileus	3(8.57)	II
DGE	4(11.43)	II
Hemorrhage	2(5.71)	II
**Post-operative pancreatic fistula**		
Biochemical leaks	2(5.71)	II
Readmissions	3(8.57)	

## DISCUSSION

The cavity of the bursa omentalis is not a closed cavity, but rather opens to the abdominal cavity via the foramen of Winslow, making it susceptible to tumor spread in advanced GCs (cT3-cT4). Theoretically, prophylactic bursectomy, excision of the peritoneal lining over the pancreas and transverse colon, may reduce tumor seeding and recurrence.^14^ In our study, tumor deposits in the bursa were seen in three (8.57%) cases, all poorly differentiated cT^3^-T^4^ lesions, suggesting bursectomy may be meaningful in such cases. Notably, both recurrences (5.71%) in our study were gastric bed recurrences in bursa-positive patients. Other studies have shown similar bursa involvement. Kayaalp et al. reported a 10% positive rate,^14^ and Yamamura et al. reported 10.29% bursa cytology positivity.^15^ They detected CEA or CK20 mRNA in peritoneal washes from the bursa and other sites, suggesting that bursectomy may eliminate a common site of peritoneal spread.^15^

D2 gastrectomy with bursectomy was historically considered the standard surgical procedure for advanced gastric cancer. However, concerns over safety and unclear oncologic benefits have led to debate. Excision of a layer of peritoneum overlying the transverse colon and pancreas, including the anterior pancreatic capsule, increases the risk of intraoperative and postoperative complications. Still, with experienced surgeons, it can be performed safely. A Japanese RCT by Imamura et al. found no significant difference in mortality (0.95%) or morbidity (14.3%) between bursectomy and non-bursectomy groups.^16^ In our study, the overall complication rate was 40%, though serious complications like hemorrhage occurred in only two (5.71%) patients.

Pancreas-specific complications, such as biochemical leaks, occurred in two (5.71%) patients in our study, with no clinically relevant pancreatic fistula. Biochemical leaks can be seen in up to 10% of patients after bursectomy.^17^ The low incidence of this is mainly attributed to meticulous dissection and the experience of the surgeons. POPF was also not statistically significant between non-bursectomy and bursectomy groups (5% vs 15%) in phase III, open-label RCT conducted in Japan and in a retrospective study by Kochi et.al. (25.6% vs 24%).^18, 19^ Delayed gastric emptying and ileus were seen in four (11.43%) and three (8.57%), respectively. It’s higher than in Imamura’s study (4.83%), possibly due to adhesions from the skeletonized mesocolon and pancreas.^16^

The survival benefit of bursectomy remains controversial. In our follow-up of at least 18 patients for more than six months, there was no mortality. Yoshikawa et al. and Kochi et al. found no survival advantage in retrospective studies.^19,20^ Data analysis of the Japanese national data registry for gastric cancer revealed that 10.7% of peritoneal recurrences occurred among sub-serosal cancers after curative gastrectomy.^21^ Blouhos et.al. concluded that bursectomy shouldn’t be considered as a futile procedure until definitive data are extracted from a large-scale study.^22^ A large Japanese phase III RCT (1,503 patients) also found no difference in five-year survival (76.7% vs. 76.9%). Thus, current evidence supports D2 gastrectomy with omentectomy, without bursectomy, as the standard for resectable cT3-T4a gastric cancers.^18^

## CONCLUSION

There are tumor deposits in the bursa, and bursectomy doesn’t increase the post-operative complications.

## Data Availability

The data are available from the corresponding author upon reasonable request
